# ﻿Taxonomic study of *Collybiopsis* (Omphalotaceae, Agaricales) in the Republic of Korea with seven new species

**DOI:** 10.3897/mycokeys.88.79266

**Published:** 2022-03-30

**Authors:** Ji Seon Kim, Yoonhee Cho, Ki Hyeong Park, Ji Hyun Park, Minkyeong Kim, Chang Sun Kim, Young Woon Lim

**Affiliations:** 1 School of Biological Sciences and Institute of Microbiology, Seoul National University, Seoul 08826, Republic of Korea Seoul National University Seoul Republic of Korea; 2 Water Supply and Sewerage Research Division, National Institute of Environmental Research, Incheon 22689, Republic of Korea National Institute of Environmental Research Incheon Republic of Korea; 3 Microorganism Resources Division, National Institute of Biological Resources, Incheon, Republic of Korea National Institute of Biological Resources Incheon Republic of Korea; 4 Forest Biodiversity Division, Korea National Arboretum, Pocheon-si 11186, Republic of Korea Korea National Arboretum Pocheon-si Republic of Korea

**Keywords:** *Collybia*, gymnopoid, *Gymnopus*, ITS, *Marasmiellus*, marasmioid, nrLSU

## Abstract

*Collybiopsis* is a genus of the gymnopoid/marasmioid complex of the family Omphalotaceae. The classification system of *Collybiopsis* has recently undergone large changes through molecular approaches. The new classification system has not been applied for *Collybiopsis* in the Republic of Korea, and general research on this genus was also lacking. In this study, we analyzed the *Collybiopsis* species in the Republic of Korea by assessing all gymnopoid/marasmioid specimens collected nationwide for ten years by combining morphological approaches and multilocus (ITS + nrLSU) phylogenetic analysis. We thus confirmed that 16 species of *Collybiopsis* are present in the Republic of Korea, including two previously unreported species (*Co.nonnulla* and *Co.dichroa*) and seven new species (*Co.albicantipes***sp. nov.**, *Co.clavicystidiata***sp. nov.**, *Co.fulva***sp. nov.**, *Co.orientisubnuda***sp. nov.**, *Co.subumbilicata***sp. nov.**, *Co.undulata***sp. nov.**, and *Co.vellerea***sp. nov.**). A thorough examination of the *Collybiopsis* suggested that it is difficult to distinguish or identify the species based on morphological characteristics only; a combined molecular approach is needed for accurate identification. The *Collybiopsis* database of the Republic of Korea is updated, and information on the new species is provided. Five new combinations from *Marasmiellus* to *Collybiopsis* are also proposed (*Co.istanbulensis***comb. nov.**, *Co.koreana***comb. nov.**, *Co.omphalodes***comb. nov.**, *Co.pseudomphalodes***comb. nov.**, and *Co.ramuliciola***comb. nov.**).

## ﻿Introduction

*Collybiopsis*Earle (1909) is a genus of gymnopoid/marasmioid mushrooms belonging to the family Omphalotaceae Bresinsky (Earle 1909; Petersen and Hughes 2021). Species of *Collybiopsis* are characterized by collybioid, gymnopoid, marasmielloid, omphalioid, and pleurotoid basidiomata; free to decurrent lamellae; a central to eccentric, insititious to subinsititious stipe; ellipsoid to oblong, inamyloid, and hyaline basidiospores with white sporeprints; presence of caulocystidia; and coralloid or diverticulate terminal elements of pileipellis (Murrill 1915; Singer 1973; Antonín and Noordeloos 1993; Retnowati 2018; Oliveira et al. 2019). Owing to its relatively uncharacteristic basidiocarp and little variation in morphological characteristics, most gymnopoid/marasmioid species were previously placed within the genus *Collybia*Staude (1857) and *Marasmius* Fr. (1835) before molecular identification was introduced actively to taxonomy. However, recent molecular studies have clarified the phylogenetic relationship of gymnopoid/marasmioid species belonging to the family Omphalotaceae and family Marasmiaceae Roze ex Kühner (Wilson and Desjardin 2005; Oliveira et al. 2019).

Initial molecular studies have segregated *Collybia* and *Marasmius* and some species of both genera transferred into several genera such as *Gymnopus* Roussel, *Marasmiellus* Murril, *Rhodocollybia* Singer, etc. (Moncalvo et al. 2002; Mata et al. 2004b; Mata et al. 2004c; Wilson and Desjardin 2005; Hughes et al. 2010; Oliveira et al. 2019; Petersen and Hughes 2017, 2021). Five *Collybia* sections (*Iocephalae* Halling, *Levipedes* Quél, *Striipedes* Quél, *Subfumosae* Singer, and *Vestipedes* Quél) were subsumed into *Gymnopus* sensu lato (s.l.) (Mata et. al., 2004c). However, *Gymnopus* s. l. is polyphyletic, and there has been much debate on the delimitation of this genus (Mata et al, 2004c; Wilson and Desjardin 2005; Mata et al. 2006; Oliveira et al. 2019; Petersen and Hughes 2016). Prior to this debate, a monophyletic genus, *Marasmiellus* sensu stricto (s. str.), was proposed (Wilson and Desjardin 2005), with *Marasmiellusjuniperinus* Murrill as the monotype species (Wilson and Desjardin 2005; Sandoval-Leiva et al. 2016; Oliveira et al. 2019). A recent study showed that if judged congeneric, *Collybiopsis*Earle (1909) has priority over *Marasmiellus*Murrill (1915) based on the nomenclature rule (Petersen and Hughes 2021). Hereupon, *Collybiopsis* has been redefined based on the type species, *Collybiopsisramealis* Earle, with at least 44 closely related species (Petersen and Hughes 2021). All species of *Collybiopsis* and some species of Gymnopussect.Vestipedes, as well as some species of *Marasmiellus*, are included in the genus *Collybiopsis* (Petersen and Hughes 2021).

*Collybiopsis* is morphologically similar and phylogenetically close to *Gymnopus* (Desjardin et al. 1999; Mata 2002; Dutta et al. 2015). Both genera are reported to be distinguishable through like types of the terminal element of pileipellis, attachment of lamellae, the character of stipe, basidiospores, and cheilocystidia. However, as the characteristics of each genus cannot be seen as absolute because exceptions exist, and some characteristics overlap, it is difficult to distinguish *Collybiopsis* from *Gymnopus* solely on morphology. Furthermore, the morphological characteristics of their basidiomata vary greatly depending on the environment and developmental stage. Therefore, molecular data play an important role in distinguishing these genera (Antonín and Herink 1999; Hughes et al. 2014; Hughes and Petersen 2015).

Although there have been many taxonomic changes for gymnopoid/marasmioid species, these changes have not been reflected in the gymnopoid/marasmioid species in the Republic of Korea. Since the first report of *Collybiopsisconfluens* (Pers.) R.H. Petersen, as its previous name *Collybiaconfluens* Fr. (Kaburagi 1940), nine current *Collybiopsis* species have been reported until recently (National list of species of Korea 2020). However, they were identified and classified as *Collybia*, *Gymnopus*, and *Marasmiellus* based on their macroscopic morphological features. Owing to the uncertain placement of previous morphologically identified collybioid collections, it was necessary to re-examine Korean collections of collybioids and marasmioids based on molecular data. In this study, we investigated gymnopoid/marasmioid specimens collected over 10 years and deposited in three Korean herbaria based on their molecular analysis. As a result, we provide a list of *Collybiopsis* species in the Republic of Korea with seven new species.

## ﻿Methods

### ﻿Collections of specimens

A total of 372 specimens deposited in three Korean fungal herbarium – Seoul National University Fungus Collection (**SFC**), Korea National Arboretum (**KA**), and the National Institute of Biological Resources (**NIBR**) – were used in this study. The specimens were collected from 2012 to 2021 and stored in dried condition. All specimens were identified based on their morphological characteristics by each herbarium. The collection information (e.g. collection date, collection site, collector, etc.) and the notes of fresh basidiomata of each specimen were provided from each herbarium.

### ﻿Molecular analysis

Genomic DNA was extracted from each specimen using a modified CTAB DNA extraction protocol (Rogers and Bendich 1994). The primer set ITS1F/ITS4B (Gardes and Bruns 1993) was used to amplify the internal transcribed spacer (ITS) region for all specimens, and the primer set LR0R/LR5 (Vilgalys and Hester 1990; Rehner and Samuels 1994) was used to amplify the nuclear large subunit ribosomal RNA (nrLSU) region. PCR was conducted by a C1000 thermal cycler (Bio-Rad, Richmond, CA, USA) using AccuPower PCR master premix (Bioneer Co., Daejeon, the Republic of Korea). PCR conditions for ITS and nrLSU region were: 5 min initial denaturation at 95 ˚C followed by 35 cycles of 40 s at 95 ˚C, 40 s at 55 ˚C and 60 s at 72 ˚C with a final extension step for 7 min at 72 ˚C. The amplifications of the PCR products were verified by visualization using 1% agarose gels with EcoDye DNA staining solution (SolGent Co., Daejeon, the Republic of Korea). The PCR products were purified using the ExpinTM PCR Purification Kit (GeneAll Biotechnology, Seoul, the Republic of Korea) following the manufacturer’s instructions. The purified PCR amplicons were sequenced using an ABI Prism 3700 Genetic Analyzer (Life Technologies, Gaithersburg, MD, USA) at Macrogen (Seoul, the Republic of Korea).

**Figure 1.**
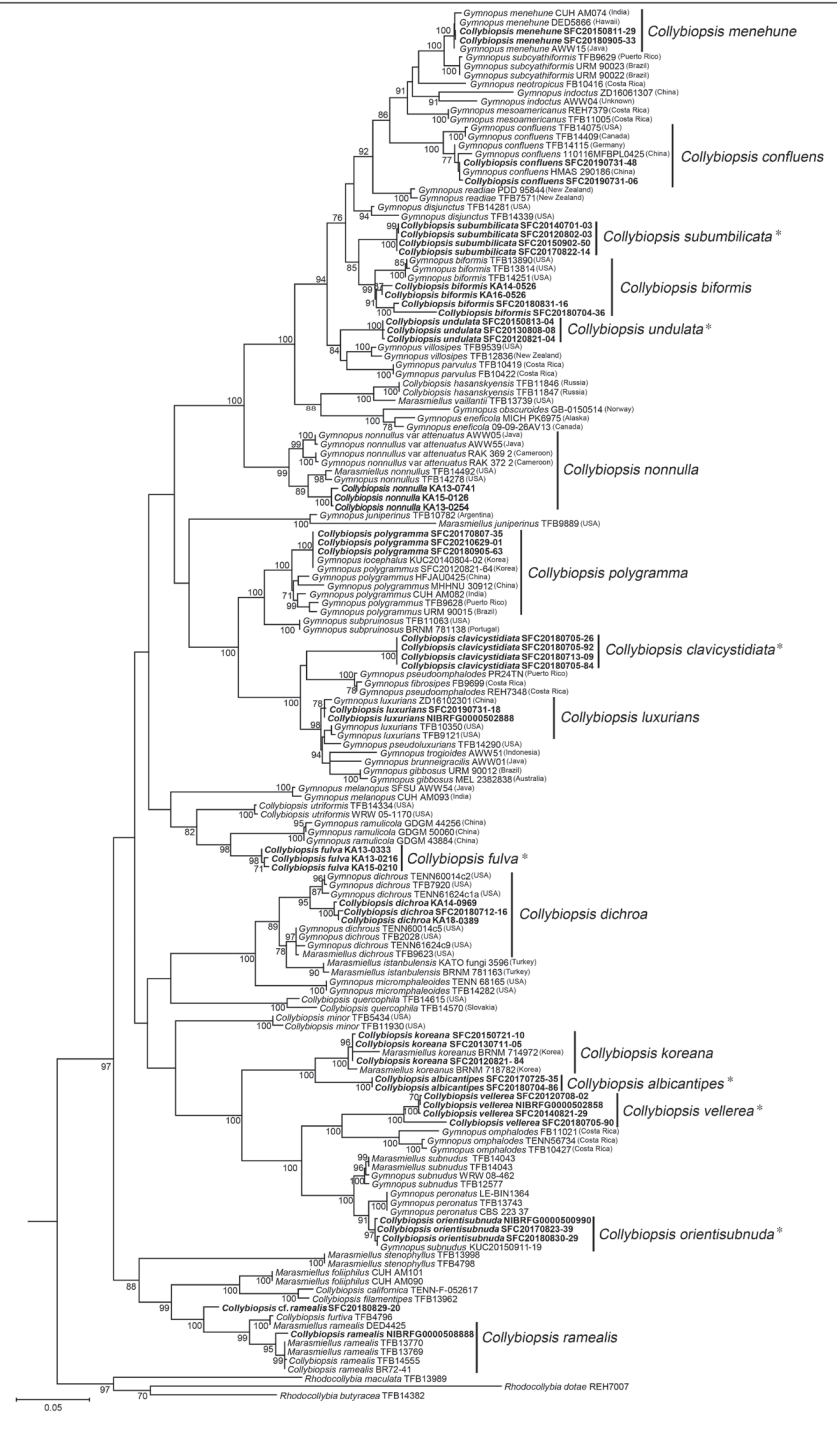
Phylogenetic tree based on maximum likelihood analysis using combined sequence data of ITS and nrLSU. ML bootstrap values greater than 70% are indicated at the nodes. *Collybiopsis* species that were newly sequenced in this study are represented in bold. Species with an asterisk are those proposed as new species.

All sequences generated in this study were proofread using MEGA version 7 (Kumar et al. 2016). The sequences used for analyses were deposited in GenBank (Table 1). We then selected the closely related sequences from NCBI databases mainly referred to Oliveira et al. (2019) and Petersen and Hughes (2021). After retrieving all published ITS and nrLSU sequences of all *Collybiopsis* species in GenBank, phylogenetic analyses were performed together with new sequences generated from specimens. The sequences were respectively aligned for each loci using Multiple Alignment Fast Fourier Transform (MAFFT ver. 7) with the L-NSI-I option algorithm (Katoh and Standley 2013). The aligned sequence data were manually checked and edited. The final sequence of each specimen was created as a concatemer by manually attaching the aligned sequences of the two loci. Maximum likelihood (ML) phylogenetic tree was constructed on the CIPRES Science Gateway (Miller et al. 2012) using the GTR+GAMMA model with 1000 bootstrap replicates. *Rhodocollybiabutyraceae* Lennox (TFB14382), *Rhodocollybiadotae* JL Mata and Halling (REH7007), and *Rhodocollybiamaculate* Singer (TFB13989) were used as outgroups (Oliveira et al. 2019). Bootstraps higher than 70% were considered to support a clade and are shown in the tree (Figure 1).

**Table 1.** Information about the *Collybiopsis* specimens and published *Collybiopsis* sequences used in phylogenetic analysis. Species with an asterisk are those proposed as new species. Sequences newly produced in this study are presented in bold.

Organisms	Specimen	Collection Date	Location	GenBank Accession Number	
				ITS	nrLSU
Collybiopsisalbicantipes*	**SFC20170725-35**	25.7.2017	Yeosu-si, Jeollanam-do, the Republic of Korea	**OL467272**	**OL462811**
	**SFC20180704-86**	4.7.2018	Jindo-gun, Jeollanam-do, the Republic of Korea	**OL467273**	**OL462812**
*Co.biformis*	TFB14251		USA: Tennessee, GSMNP	KJ416245	KJ189567
	TFB13890		USA: North Carolina	KJ416248	KJ189570
	TFB13814		USA: Tennessee	KJ416249	KJ189569
	**KA14-0526**	15.7.2014	Suncheon, Jeollanam-do, the Republic of Korea	**OL467227**	**OL462784**
	**KA16-0526**	13.7.2016	Sinan-gun, Jeollanam-do, the Republic of Korea	**OL467228**	**OL462785**
	**SFC20180704-36**	4.7.2018	Wando-gun, Jeollanam-do, the Republic of Korea	**OL467229**	**OL462789**
	**SFC20180831-16**	31.8.2018	Jindo-gun, Jeollanam-do, the Republic of Korea	**OL467230**	**OL462790**
*Co.brunneigracilis*	AWW01		Java/Bali	AY263434	AY639412
*Co.californica*	TFB5787		Canada: British Columbia	MN413338	
Co.clavicystidiata*	**SFC20180705-26**	5.7.2018	Haenam-gun, Jeollanam-do, the Republic of Korea	**OL467250**	**OL462816**
	**SFC20180705-84**	5.7.2018	Jindo-gun, Jeollanam-do, the Republic of Korea	**OL467252**	**OL462817**
	**SFC20180705-92**	5.7.2018	Jindo-gun, Jeollanam-do, the Republic of Korea	**OL467253**	**OL462818**
	**SFC20180713-09**	13.7.2018	Gwanak-gu, Seoul, the Republic of Korea	**OL467251**	**OL462819**
*Co.confluens*	**SFC20190731-06**	31.7.2019	Taebaek-si, Gangwon-do, the Republic of Korea	**OL467237**	**OL462797**
	**SFC20190731-48**	31.7.2019	Taebaek-si, Gangwon-do, the Republic of Korea	**OL467238**	**OL462798**
	TFB14115		Germany, Thuringia	KP710292	KJ189578
	110116MFBPL0425		China	MW554401	
	HMAS 290186		China	MK966541	
*Co.confluens* ssp. americana	TFB14409		Canada: New Brunswick	KP710278	KJ189585
	TFB14075		USA: North Carolina	KP710281	KJ189581
*Co.dichroa*	**KA14-0969**	19.8.2014	Hwasun-gun, Jeollanam-do, the Republic of Korea	**OL467254**	**OL462799**
	**KA18-0389**	10.7.2018	Cheongdo-gun, Gyeongsangbuk-do, the Republic of Korea	**OL467255**	**OL546541**
	**SFC20180712-16**	12.7.2018	Gwangju, Gyeonggi-do, the Republic of Korea	**OL467256**	**OL462800**
	TFB9623		USA: North Carolina	MW396865	MW396865
	TENN60014c2		USA: Tennessee, GSMNP	JF313671	
	TFB7920		USA	DQ450007	
	TENN61624c1a		USA: Tennessee, GSMNP	JF313678	
	TFB2028		USA	DQ450008	
	TENN61624c9		USA: Tennessee, GSMNP	JF313692	
*Co.disjuncta*	TFB14339		USA: Connecticut	NR_137865	
	TFB14281		USA: Mississippi	KJ416253	KY019643
*Co.eneficola*	09-09-26AV13		Canada: Newfoundland	NR_137613	NG_059502
	MICH:PK6975		Alaska	KP710270	KP710304
*Co.fibrosipes*	FB9699		Costa Rica	AF505763	
*Co.filamentipes*	TFB13962		USA: Tennessee	MN897832	MN897832
*Co.foliiphila*	CUH AM090		India	NR_154176	NG_060320
	CUM AM101		India	KP317638	KP317636
Co.fulva*	**KA13-0216**	19.6.2013	Geochang-gun, Gyeongsangnam-do, the Republic of Korea	**OL467257**	**OL462793**
	**KA13-0333**	10.7.2013	Pocheon-si, Gyeonggi-do, the Republic of Korea	**OL467258**	**OL462794**
	**KA15-0210**	21.7.2015	Pocheon-si, Gyeonggi-do, the Republic of Korea	**OL467259**	**OL462795**
*Co.furtiva*	TFB4796		USA: Georgia	MN413343	MW396879
*Co.gibbosa*	MEL:2382838		Australia	KP012713	KP012713
	URM 90012		Brazil	KY061202	KY061202
*Co.hasanskyensis*	TFB11846		Russia: Kedrovaya	MN897829	
	TFB11847		Russia	MN897830	
*Co.indoctus*	AWW04		Unknown	AY263439	
*Co.istanbulensis*	KATO Fungi 3596		Turkey	KX184795	KX184796
	BRNM 781163		Turkey	KY250435	
*Co.juniperina*	TFB9889		USA: Louisiana	AY256708	KY019637
	TFB10782		Argentina: Missiones	KY026661	KY026661
*Co.koreana*	**SFC20120821-84**	21.8.2012	Boryeong-si, Chungcheongnam-do, the Republic of Korea	**OL467269**	**OL546545**
	**SFC20130711-05**	11.7.2013	Pyeongchang-gun, Gangwon-do, the Republic of Korea	**OL467270**	**OL462801**
	**SFC20150721-10**	21.7.2015	Inje-gun, Gangwon-do, the Republic of Korea	**OL467271**	**OL462802**
	BRNM 714972		Korea	GU319113	GU319117
	BRNM 718782		Korea	GU319114	GU319118
*Co.luxurians*	**NIBRFG0000502888**	4.9.2018	Ongjin-gun, Incheon, the Republic of Korea	**OL467248**	**OL462803**
	**SFC20190731-18**	31.7.2019	Taebaek-si, Gangwon-do, the Republic of Korea	**OL467249**	**OL462804**
	TFB10350		USA: North Carolina	AY256709	AY256709
	ZD16102301		China	MN523270	
	TFB9121		USA: Louisiana	KY026649	KY026649
*Co.melanopus*	AWW54		Java/Bali	NR_137539	NG_060624
	CUH AM093		India	KM896875	KP100305
*Co.menehune*	**SFC20150811-29**	11.8.2015	Guri-si, Gyeonggi-do, the Republic of Korea	**OL467235**	**OL462805**
	**SFC20180905-33**	5.9.2018	Anyang City, Gyeonggi Province, the Republic of Korea	**OL467236**	**OL462806**
	SFSU: DED5866		Hawaii	AY263426	
	CUH:AM074		India	KJ778753	KP100302
	SFSU-AWW15		Java/Bali	AY263443	AY639424
*Co.mesoamericana*	TFB11005		Costa Rica	DQ450035	KY019632
	REH7379		Costa Rica	AF505768	
*Co.micromphaleoides*	TENN 68165				
	TFB14282				
*Co.minor*	TFB11930		USA: Tennessee, GSMNP	MN413334	MW396880
	TFB5434		USA: South Carolina	MW396872	MW396872
*Co.neotropica*	TFB10416		Costa Rica	AF505769	
*Co.nonnulla*	**KA13-0254**	20.6.2013	Geochang-gun, Gyeongsangnam-do, the Republic of Korea	**OL467242**	**OL462820**
	**KA13-0741**	21.8.2013	Geochang-gun, Gyeongsangnam-do, the Republic of Korea	**OL467243**	**OL462807**
	**KA15-0129**	14.7.2015	Gangneung-si, Gangwon-do, the Republic of Korea	**OL467244**	**OL462808**
	TFB14492		USA: Mississippi	KY026699	KY026699
	TFB14278		USA: Mississippi	KY026701	KY026701
*Co.nonnulla* var. attenuatus	AWW05		Java/Bali	AY263445	AY639426
	AWW55		Java/Bali	AY263446	
	RAK369.2		Cameroon	MN930621	
	RAK372.2		Cameroon	MN930622	
*Co.obscuroides*	GB-0150514		Norway: Svalbard	KX958399	KX958399
*Co.omphalodes*	FB11021		Costa Rica	AF505761	
	TFB 10427		Costa Rica	DQ450011	
	TFB 10022		Costa Rica	AY256700	
Co.orientisubnuda*	**NIBRFG0000500990**	19.7.2016	Ulleung-gun, Gyeongsangbuk-do, the Republic of Korea	**OL467262**	**OL546546**
	**SFC20170823-39**	23.8.2017	Hapcheon-gun, Gyeongsangnam-do, the Republic of Korea	**OL467263**	**OL546547**
	**SFC20180830-29**	30.8.2018	Hapcheon-gun, Gyeongsangnam-do, the Republic of Korea	**OL467264**	**OL462796**
(as *Gymnopussubnudus*)	KUC20150911-19		Korea	KX513748	
*Co.parvula*	TFB10419		Costa Rica	DQ450060	
	TFB10422		Costa Rica	AF505774	
*Co.peronata*	TFB13743		Belgium	KY026677	KY026677
	LE-Bin1364		Russia	KY026755	KY026755
	CBS 223.37		unknown	MH855896	MH867405
*Co.polygramma*	**SFC20170807-35**	7.8.2017	Hapcheon-gun, Gyeongsangnam-do, the Republic of Korea	**OL467245**	**OL546542**
	**SFC20180905-63**	5.9.2018	Gwanak-gu, Seoul, the Republic of Korea	**OL467246**	**OL546544**
	**SFC20210629-01**	29.6.2021	Gwanak-gu, Seoul, the Republic of Korea	**OL467247**	**OL546543**
	PR2542TN		Puerto Rico	DQ450028	
	CUH:AM082		India	KJ778752	KP100303
	URM 90015		Brazil: Amapa	KY074640	KY088275
	MHHNU 30912		China	MK214392	
	TFB9628		Puerto Rico	DQ450028	
	SFC20120821-64		Korea	KJ609162	
	HFJAU 0425		China: Jiangxi	MN258643	
(as *Gymnopusiocephalus*)	KUC20140804-02		Korea	KX513745	
*Co.pseudoluxurians*	TFB14290		USA: Mississippi	NR_137863	
*Co.pseudomphalodes*	REH7348		Costa Rica	AF505762	
	PR24TN		Puerto Rico	AY842957	
*Co.quercophila*	TFB14570		Slovakia	KY026728	KY026728
	TFB14615		USA: California	KY026736	KY026736
*Co.ramealis*	**NIBRFG0000508888**	29.7.2020	Jeongseon-gun, Gangwon-do, the Republic of Korea	**OL467260**	**OL546549**
	TFB13769		Belgium: Couvin	MN413345	MN413345
	TFB13770		Belgium: Couvin	MN413346	MW396882
	DED4425		USA: North Carolina	DQ450031	AF042650
	TFB14555		Slovakia	MW405779	MW396884
	BR 72_41		Belgium	MW396875	MW396875
*Co.ramulicola*	GDGM 43884		China	KU057798	
	GDGM 44256		China	KU321529	
	GDGM 50060		China	KU321530	
*Co.readiae*	TFB7571		New Zealand	DQ450034	
	PDD:95844		New Zealand	HQ533036	
*Co.stenophylla*	TFB13998		USA: Tennessee,	MN413331	MW396886
	TFB4798		USA: Georgia	MN413330	MW396887
*Co.subcyathiformis*	TFB9629				
	URM 90023		Brazil: Para	KY404982	KY404982
	URM 90022		Brazil: Para	KY404983	KY404983
*Co.subnuda*	TFB12577		USA: Tennessee, GSMNP	KY026667	FJ750262
	WRW 08-462		USA: West Virginia	KY026765	KY026765
	TFB14043		USA: North Carolina	MW396876	MW396876
*Co.subpruinosus*	BRNM781138		Portugal: Madeira	MK646034	
	TFB11063		USA	DQ450025	
Co.subumbilicata*	**SFC20120802-03**	2.8.2012	Goseong-gun, Gangwon-do, the Republic of Korea	**OL467231**	**OL462786**
	**SFC20140701-03**	1.7.2014	Inje-gun, Gangwon-do, the Republic of Korea	**OL467232**	**OL462787**
	**SFC20150902-50**	2.9.2015	Ulleung-gun, Gyeongsangbuk-do, the Republic of Korea	**OL467234**	**OL546540**
	**SFC20170822-14**	22.8.2017	Ulleung-gun, Gyeongsangbuk-do, the Republic of Korea	**OL467233**	**OL462788**
*Co.trogioides*	AWW51		Indonesia	AY263428	AY639431
Co.undulata*	**SFC20120821-04**	21.8.2012	Boryeong-si, Chungcheongnam-do, the Republic of Korea	**OL467239**	**OL462813**
	**SFC20130808-08**	8.8.2013	Sangju-si, Gyeongsangbuk-do, the Republic of Korea	**OL467240**	**OL462814**
	**SFC20150813-04**	13.8.2015	Goyang-si, Gyeonggi-do, the Republic of Korea	**OL467241**	**OL462815**
*Co.utriformis*	TFB14334h1		USA: Connecticut	KY026708	KY026708
	WRW05-1170		USA: West Virginia	KY026764	KY026764
Co.vellerea*	**NIBRFG0000502858**	4.9.2018	Ongjin-gun, Incheon, the Republic of Korea	**OL467265**	**OL462791**
	**SFC20120708-02**	8.7.2012	Seosan-si, Chungcheongnam-do, the Republic of Korea	**OL467266**	**OL462809**
	**SFC20140821-29**	21.8.2014	Gwanak-gu, Seoul, the Republic of Korea	**OL467267**	**OL462810**
	**SFC20180705-90**	5.7.2018	Jindo-gun, Jeollanam-do, the Republic of Korea	**OL467268**	**OL462792**
*Co.vallianti*	TFB13739		USA: Tennessee, GSMNP	KY026676	KY026676
*Co.villosipes*	TFB9539		USA	DQ450058	
	TFB12836		New Zealand: Fiordland	KJ416255	FJ750264
Collybiopsiscf.ramealis	**SFC20180829-20**	29.8.2018	Shinan-gun, Jeollanam-do, the Republic of Korea	**OL467261**	**OL546548**
*Rhodocollybiabutyracea*	TFB 14382		Canada: New Brunswick	KY026716	KY026716
*Rhodocollybiadotae*	REH7007		Costa Rica	AF505758	
*Rhodocollybiamaculata*	TFB 13989		USA: Mississippi	KY026688	KY026688

### ﻿Morphological observation

All specimens were preliminarily observed and macro/micro-structures of two to four representative specimens, which were in the best condition among the specimens, were presented in figures. Photographs and notes of fresh basidiomata taken at the time of collection were used for macro-morphological description. For micro-morphological observations, tissues of dried specimens were rehydrated in 5% (w/v) KOH and mounted in Congo red solution (Clémençon 1973) and Melzer’s reagent. The observation was performed by using a Nikon Eclipse 80i optical microscope (Nikon, Tokyo, Japan) at 20 × to 1000 × magnification. More than thirty basidiospores and more than twenty other microstructures (e.g., basidia, cheilocystidia, etc.) were measured to analyze the microstructures based on the microscopic pictures of specimens stained with Congo red. The Methuen Handbook of Colour (Kornerup and Wanscher 1978) was used for color indications. The following abbreviations and acronyms were used: ***Co.*** = *Collybiopsis*; ***G*** = *Gymnopus*; ***Ma*** = *Marasmiellus*; **L** = the number of complete lamellae; **l** = the number of lamellulae tiers between neighboring complete lamellae; and **Q** = the values of the length divided by the width of basidiospores (Petersen and Hughes 2021; Ryoo et al. 2020).

## ﻿Results

Through ITS sequence analysis of 372 gymnopoid/marasmioid specimens, 201 specimens were confirmed to belong to *Collybiopsis*. The remaining 160 specimens were identified as members of the following genera: *Gymnopus*, *Marasmius*, or *Rhodocollybia* and were excluded from this study. A total of 201 specimens were segregated into 16 putative taxa based on ITS phylogenetic analyses (Table 2). To confirm the species’ identity and to infer the phylogenetic relationships within *Collybiopsis*, the nrLSU region was amplified and sequenced from 47 representative specimens of 16 taxa (Table 1). The final phylogenetic analyses were conducted with datasets of two loci from 16 *Collybiopsis* species (Table 1). In ML analysis, 178 multigene sequences (110 for ITS and 68 for nrLSU) were retrieved from GenBank and used. The adjusted alignments comprised 535 to 794 bases for ITS and 324 to 904 bases for nrLSU. The phylogenetic analysis results of the two combined loci revealed that *Collybiopsis* specimens from the Republic of Korea were identified as 16 taxa (Fig. 1).

**Table 2.** Identification information of Korean *Collybiopsis* specimens confirmed in the study. Scientific names in bold indicate new species.

Species	Specimen Number				
***Co.albicantipes***	SFC20170725-35	SFC20180704-86			
*Co.biformis*	NIBRFG0000502789	KA14-0259	KA14-0526	KA14-0917	KA14-0924
	KA16-0307	KA16-0371	KA16-0526	KA18-0657	KA18-0673
	SFC20140724-41	SFC20160719-42	SFC20180704-36	SFC20180706-05	SFC20180831-13
	SFC20180831-16				
***Co.clavicystidiata***	KA14-0667	KA14-0724	KA15-0211	KA17-0287	KA17-0369
	KA18-0282	KA18-0353	SFC20180705-84	SFC20180705-92	SFC20180706-04
	SFC20180713-09				
*Co.confluens*	NIBRFG0000508913	NIBRFG0000508991	KA16-0696	KA18-0338	SFC20150626-26
	SFC20190731-06	SFC20190731-32	SFC20190731-34	SFC20190731-48	
*Co.dichroa*	KA14-0969	KA17-0344	SFC20180706-60	SFC20180712-16	
***Co.fulva***	KA13-0215	KA13-0216	KA13-0333	KA13-0357	KA14-0168
	KA14-0386	KA14-0666	KA14-0691	KA15-0210	KA16-0425
	KA16-0428	KA17-0388	KA17-0596	KA18-0233	KA18-0241
*Co.koreana*	SFC20120821-84	SFC20150702-25	SFC20170713-06	SFC20180704-17	
*Co.luxurians*	NIBRFG0000502888	SFC20190731-18	SFC20190731-08	SFC20190730-36	SFC20180907-105
	SFC20180905-86	SFC20180905-43	KA18-0321	KA14-0579	
*Co.menehune*	NIBRFG0000502876	KA13-0887	KA14-0494	KA14-0510	SFC20150811-29
	SFC20160719-15	SFC20180905-33			
*Co.nonnulla*	KA13-0254	KA13-0741	KA15-0129		
***Co.orientisubnuda***	SFC20170823-39	SFC20170708-14	SFC20150902-01	SFC20150820-59	SFC20150820-01
	SFC20150811-48	SFC20150701-100	QM20200911-57	QM20200911-52	KA17-0787
	KA17-0600	KA16-1154	KA16-0925	KA16-0902	KA16-0780
	KA16-0724	KA15-0179	KA14-0985	KA13-1225	F20200730-24
	F20200701-11	F20200630-30	F20180904KCM21	F20160719-12	
*Co.polygramma*	KA13-0506	KA13-0956	KA13-1101	KA13-1333	KA14-0904
	KA14-1089	KA14-1092	KA18-0115	KA18-0724	QM20200721-07
	NIBRFG0000508098	NIBRFG0000508059	NIBRFG0000508089	SFC20170712-08	SFC20170807-35
	SFC20170822-66	SFC20180905-49	SFC20180905-63		
*Co.ramealis*	SFC20130711-05				
***Co.subumbilicata***	KA13-1214	KA15-0173	KA15-0185	KA15-0787	SFC20120802-03
	SFC20140701-03	SFC20150902-50	SFC20170822-14	SFC20210623-03	
***Co.undulata***	KA17-0335	KA18-0651	KA18-0651	SFC20120821-04	SFC20130808-08
	SFC20150715-24	SFC20150813-04			
***Co.vellerea***	KA14-0132	KA14-0163	KA14-0196	KA14-0245	KA14-0397
	KA14-0412	KA14-0446	KA14-0447	KA14-0474	KA14-0725
	KA14-0734	KA14-0735	KA14-0774	KA14-0787	KA14-1005
	KA14-1061	KA14-1147	KA14-1349	KA14-1426	KA14-1475
	KA14-1555	KA14-1558	KA15-0213	KA15-0215	KA15-0473
	KA15-0485	KA15-0502	KA15-0527	KA15-0568	KA16-0191
	KA16-0252	KA16-0485	KA16-0783	KA16-0982	KA16-0985
	KA16-0986	KA16-0992	KA17-0368	KA17-0586	KA17-0742
	KA17-1074	KA18-0089	KA18-0139	KA18-0151	KA18-0152
	KA18-0348	KA18-0795	KA18-0836	KA18-0987	KA18-1027
	KA19-0125	SFC20120708-02	SFC20120820-02	SFC20140821-29	SFC20150630-38
	SFC20150714-01	SFC20170705-06	SFC20180705-90	SFC20180829-30	SFC20180901-01
Collybiopsiscf.ramealis	F20200729-14				

**Figure 2.**
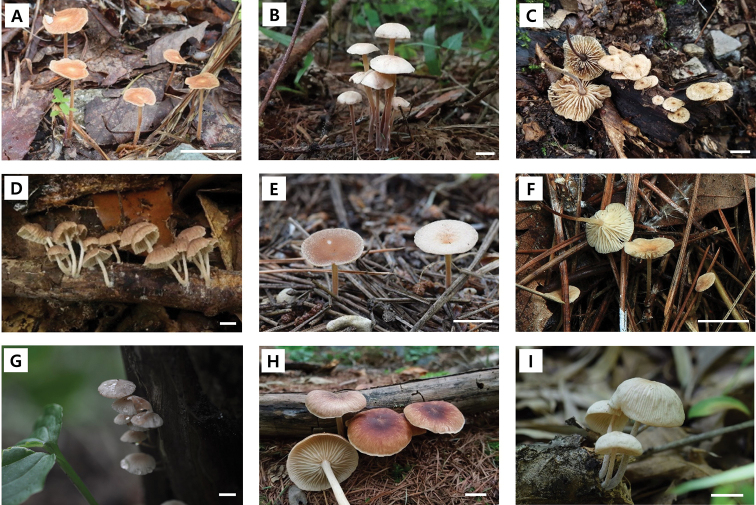
Basidiomata of the described *Collybiopsis* species in the Republic of Korea **A***Co.biformis* (SFC20180706–05) **B***Co.confluens* (SFC20190731–06) **C***Co.dichroa* (KA18–0389) **D***Co.koreana* (SFC20180704–17) **E***Co.luxurians* (SFC20190731–18) **F***Co.menehune* (SFC20150811–29) **G***Co.nonnulla* (KA13–0254) **H***Co.polygramma* (SFC20170712–08) **I***Co.ramealis* (SFC20180829–20). Scale bar: 1 cm (**A–I**).

**Figure 3.**
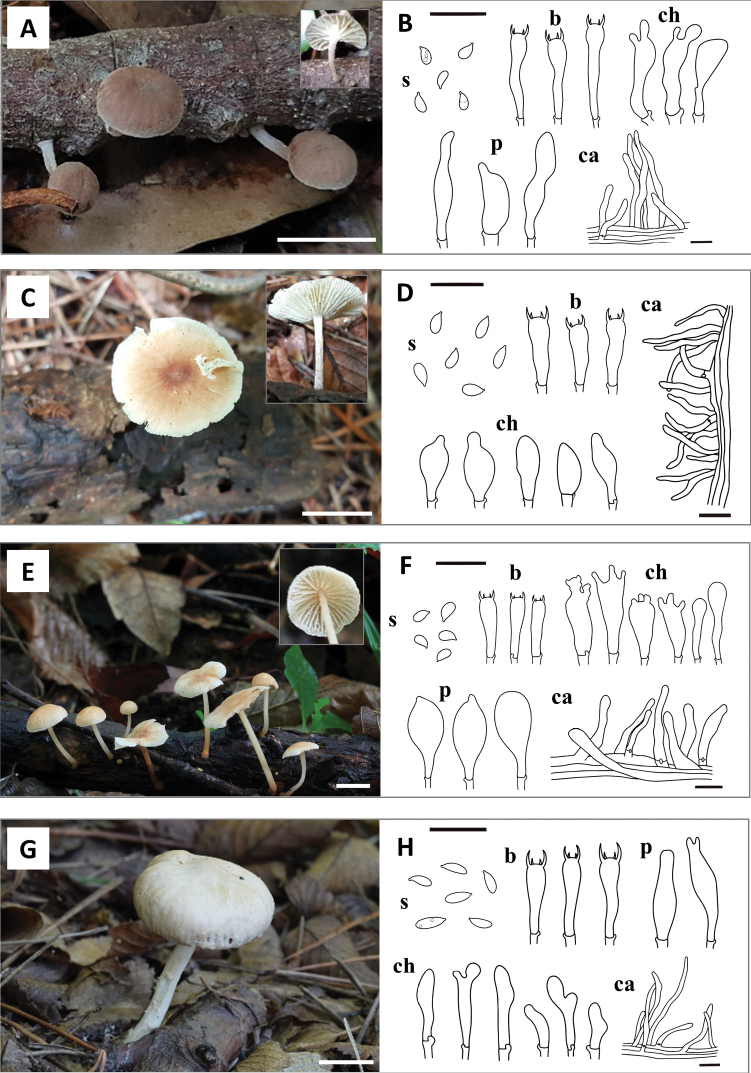
Basidiomata and microscopic characters of the four new *Collybiopsis* species **A, B***Co.albicantipes* (SFC20170725–35) **C, D***Co.clavicystidiata* (SFC20180705–84) **E, F***Co.fulva* (KA15–0210) **G, H***Co.orientisubnuda* (NIBRFG0000502862). Scale bars: 1cm (A, C, E, G); 20 *µm* (**B, D, F, H**). Abbreviations: **s** basidiospores; **b** basidia; **ch** cheilocystidia; **p** pleurocystidia; **ca** caulocystidia.

**Figure 4.**
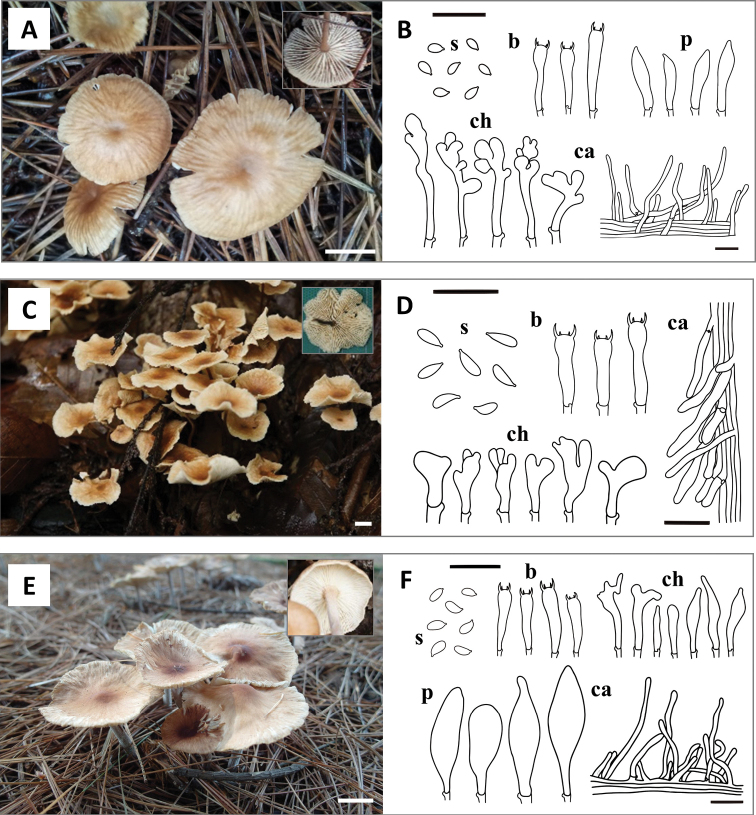
Basidiomata and microscopic characters of the three new *Collybiopsis* species **A, B***Co.subumbilicata* (SFC20120802–03) **C, D***Co.undulata* (SFC20150813–04) **E, F***Co.vellerea* (SFC20140821–29). Scale bars: 1cm (**A, C, E**); 20 *µm* (**B, D, F**). Abbreviations: **s** basidiospores; **b** basidia; **ch** cheilocystidia; **p** pleurocystidia; **ca** caulocystidia.

Of the 16 putative taxa, nine matched with previously described species – *Co.biformis* (Peck) R.H. Petersen, *Co.confluens*, *Co.dichroa* (Berk. & M.A. Curtis) Earle, *Co.luxurians* (Peck) R.H. Petersen, *Co.menehune* (Desjardin, Halling & Hemmes) R.H. Petersen, *Co.nonnulla* (Corner) R.H. Petersen, *Co.polygramma* (Mont.) R.H. Petersen, *Co.ramealis* (Bull.) Earle, and *Marasmielluskoreanus* Antonín, Ryoo & H.D. Pictures of basidiomata are shown in Fig. 2. The other seven taxa formed distinct clades and did not correspond to any known *Collybiopsis* species. Furthermore, based on the comparison with other *Collybiopsis* species, these seven species have distinct morphological characteristics, confirming that they were new to science – *Co.albicantipes* sp. nov., *Co.clavicystidiata* sp. nov., *Co.fulva* sp. nov., *Co.orientisubnuda* sp. nov., *Co.subumbilicata* sp. nov., *Co.undulata* sp. nov., and *Co.vellerea* sp. nov. Illustrations of basidiomata and micro-morphological features are shown in Figs 3 and 4.

Five species (*G.omphalodes* Halling & J.L. Mata, *G.pseudomphalodes* J.L. Mata, *G.ramulicola* T.H. Li & S.F. Deng, *Ma.istanbulensis* E. Sesli, Antonín and E.Aytaç, and *Ma.koreanus*), previously placed in GymnopussectionVestipedes, were confirmed to belong to the genus *Collybiopsis*, and we thus propose to reclassify them as *Co.omphalodes* comb. nov., *Co.pseudomphalodes* comb. nov., *Co.ramulicola* comb. nov., *Co.istanbulensis* comb. nov., and *Co.koreana* comb. nov. respectively.

### ﻿Taxonomy

#### ﻿ Collybiopsis albicantipes

Taxon classificationFungiAgaricalesMarasmiaceae

J.S. Kim & Y.W. Lim sp. nov.

BCD572AB-4B7B-5B8F-BBC1-F1FFF355ADAD

842053

Fig. 3A–B , Suppl. material 1: Fig. S1A

##### Etymology.

Epithet “*albicantipes*” refers to having a whitish base of the stipe.

##### Holotype.

The Republic of Korea, Jeollanam-do: Yeosu-si, Dolsan-eup, Hyangiram, 34°35'27"N, 127°47'55"E, alt. 183 m, 25 July 2017, Jae Young Park, Komsit Wisitrassameewong, SFC20170725–35 (GenBank accession no. ITS: OL467272; nrLSU: OL462811).

##### Diagnosis.

This species notably has hemispherical to convex, 4–23 mm pileus, distant lamellae, central to eccentric, tomentose, 5–15 × 0.5‒1.5 mm stipe with a white base; ellipsoid to ovoid, 5.8–7.4 × 2.8–4 μm basidiospores, clavate (often constricted), 25.5–34.8 × 4.8–6.7 μm basidia, broadly clavate, irregular, sometimes lobed, 26–49 × 5.4–10.6 μm cheilocystidia, and a habit of fruiting on branches.

##### Description.

Pileus: 4‒23 mm, eccentric, convex to hemispherical when young, becoming depressed and undulating with age; Surface smooth, brownish orange (5C3 to 6D4) at the center, becoming paler to the margin (4A3 to 3A2). Lamellae: distant, L = 10–16, l = 3–7, adnate, whitish to yellowish white (3A2). Stipe: 5–15 × 0.5‒1.5 mm, central to eccentric, cylindrical, tomentose, apex brownish orange (5C3) to light brown (6D4), gradually becoming paler downwards (5B2 to 6C2), with whitish basal tomentum. Basidiospores: 5.8–7.4 × 2.8–4 μm (average 5.5 × 3.2 μm), *Q* = 1.6–2.1 (mean = 1.97), ellipsoid to ovoid, amygdaliform, smooth, hyaline, non-dextrinoid, with drops. Basidia: (23) 25.5–34.8 × 4.8–6.7 (7) μm, 4-spored, clavate, often constricted. Cheilocystidia: 26–49 × 5.4–10.6 (14) μm, broadly clavate, irregular, sometimes lobed. Pleurocystidia: 25.8–56.4 (62) × 6.2–12.5 μm, clavate, subulate, sometimes lobed. Trama hyphae: cylindrical, often sub-inflated, smooth, non-dextrinoid 1.7–9 (12) μm wide. Pileipellis: a cutis made up of cylindrical, often sub-inflated, with weak annular ornamentation, 2.0‒7.5 μm wide hyphae; terminal elements adpressed, cylindrical, clavate, sometimes constricted or curved, 2.0‒5 μm wide. Stipitipellis: a cutis of cylindrical, smooth, 2.7‒9.7 (11) μm wide hyphae. Caulocystidia: 21.7–90 × 3.9–11.7 μm, cylindrical, flexuose, sometimes curved. Clamp connections: present in all tissues.

##### Other specimens examined.

The Republic of Korea, Jeollanam-do: Jindo-gun, Maenggoldo island, 34°12'21"N, 125°51'41"E, alt. 24 m, 4 July 2018, Jae Young Park, SFC20180704–86.

##### Habit and habitat.

Scattered to gregarious on the branch in mixed forest dominated by *Camelliajaponica* Linne, in summer.

##### Distribution.

The Republic of Korea.

##### Remark.

*Collybiopsisalbicantipes* is similar to *Co.ramulicola* and *Co.koreana* when comparing macro-morphological characteristics. *Collybiopsisramulicola* is distinguishable from *Co.albicantipes* by a reddish pileus, fewer and buff lamellulae (1–4), a shorter and thinner stipe (12–23 × 2–3 mm), shorter and slightly elongated basidiospores (6.6–8.4 × 3.5–4.5 μm), shorter basidia (23–27 × 3.8–5.5 μm), and shorter cheilocystidia (23–27 × 3–6 μm) (Deng et al. 2016). *Collybiopsiskoreana* differs from *Co.albicantipes* by having a larger pileus (27–60 mm), more lamellae (15–20) and lamellulae (2–3), longer and thicker stipe (14–70 × 2–3.5 μm), bigger and elongated basidiospores (7.5–10 × 4–5 μm), cheilocystidia with different shapes and sizes (25–55 × 4–10 μm), and incrustation dark brown in KOH (Antonín et al. 2010).

#### ﻿ Collybiopsis clavicystidiata

Taxon classificationFungiAgaricalesMarasmiaceae

J.S. Kim & Y.W. Lim sp. nov.

176ECE24-941E-52C3-8C9A-E50EF44C1E63

842054

Fig. 3C–D , Suppl. material 1: Fig. S1B

##### Etymology.

Epithet “*clavicystidiata*” indicates that the new species has clavate cheilocystidia.

##### Holotype.

The Republic of Korea, Jeollanam-do: Jindo-gun, Jodo-myeon, Donggeocha island, 34°23'34"N, 125°93'84"E, alt. 70 m, 05 July 2018, Jae Young Park, Tae Heon Kim, SFC20180705–84, (GenBank accession no. ITS: OL467252; nrLSU: OL462817).

##### Diagnosis.

The prominent features of this species include a greyish orange to brownish, 6–45 mm pileus, whitish lamellae, a subinstitious, tomentose, whitish, 15–26 × 1.2‒1.6 mm stipe, oblong to subcylindrical, 6.7‒9.4 × 3.1‒4.6 μm basidiospores, utriform, clavate, 20.1–37.5 × 6.8–12.2 μm cheilocystidia, and cylindrical, flexuose, irregular, 17–50 × 3.5–7 μm caulocystidia.

##### Description.

Pileus: 6–45 mm, convex to hemispherical, becoming plano-convex to flat with an uplifted margin with age; Surface smooth, dull, hygrophanous, greyish orange (6B3) to brownish (7D8 to E8) at the center, being whitish at the margin (4A2 to 6C8), being paler with age. Lamellae: subdistant, L = 20–32, l = 1–7, adnexed, white. Stipe: 15–26 × 1.2‒1.6 mm, cylindrical, tomentose, subinsititious, whitish to reddish grey (9B2). Basidiospores: 6.7‒9.4 × 3.1‒4.6 μm, average 8.13 × 3.62 μm, Q = 2–2.4 (mean = 2.26), oblong to cylindrical, smooth, hyaline, non-dextrinoid, with drops. Basidia: 18.3–30 × 4.1–8.8 μm, 4-spored, narrowly clavate, narrowly utriform, often curved. Cheilocystidia: 20.1–37.5 × 6.8–12.2 μm, utriform, clavate, sometimes with mucronate apex. Pleurocystidia: absent. Trama hyphae: cylindrical, often subinflated, smooth, branched, non-dextrinoid, 2‒12 μm wide. Pileipellis: transition between cutis and trichoderm, composed of cylindrical, with heavy annular ornamentation, 4‒12 μm wide hyphae; terminal elements adpressed to suberect, cylindrical, clavate, often incrusted (often incrusted), thin-walled, 3‒6 μm wide. Stipitipellis: a cutis of cylindrical, smooth, 2‒7 μm wide hyphae. Caulocystidia: 17–50 × 3.5–7 μm, cylindrical, flexuose, irregular or curved. Clamp connections: present in all tissues.

##### Other specimens examined.

The Republic of Korea, Jeollanam-do: Haenam-gun, Mt. Duryun, 34°29'6"N, 126°38'54"E, alt. 169 m, 5 July 2018, Young Woon Lim, Abel Severin Lupala, Jun Won Lee, SFC20180705–26. The Republic of Korea, Seoul: Gwanak-gu, Gwanak-ro 1, Seoul National University, 37° 27' 37"N, 126° 56' 59"E, alt. 80m, 13 July 2018, Jae Young Park, SFC20180713–09.

##### Habit and habitat.

Solitary to scattered on dead wood debris of conifers, in summer.

##### Distribution.

The Republic of Korea

##### Remark.

*Collybiopsisclavicystidiata* is morphologically similar to *G.omphalodes* and *Co.menehune*. *Collybiopsisomphalodes* differs in their larger pileus (2–30 mm), a darker colored stipe, smaller basidiospores (5–6 × 2.5–3 μm), and thinner hyphae in the pileipellis (5–8 μm wide). *Collybiopsismenehune* can be distinguished from *Co.clavicystidiata* by its larger pileus (8–30 mm), buff lamellae, longer stipe (15–60 mm), longer basidiospores (7.5–9.5 × 3.5–4.2 μm, Q = 2.2), and longer caulocystidia (16–67 × 3–5 μm) (Desjardin et al. 1999). *Co.clavicystidiata* is phylogenetically close to *Co.pseudomphalodes*. *Collybiopsispseudomphalodes* has relatively few references for comparison, but differences can be found in the lengths of the stipe (3–4 mm) and cheilocystidia (40 × 3 μm) when compared with *Co.clavicystidiata* (Dennis 1961).

#### ﻿ Collybiopsis fulva

Taxon classificationFungiAgaricalesMarasmiaceae

J.S. Kim & Y.W. Lim sp. nov.

FE0580F5-F50E-5D4E-A82F-26A849BA1994

842055

Fig. 3E–F , Suppl. material 1: Fig. S1C

##### Diagnosis.

This species has a pale orange to brownish-colored, 4–20 mm pileus, an orange white colored to light brownish colored, 7–30 × 0.7–1 mm stipe with pubescence, spheropedunculate, pleurocystidia, oblong to subcylindrical, 6.8–9.2 × 3.1–4.9 μm basidiospore, lobed, clavate with rostrate apex, 24.8–38.4 × 6.5–11.8 μm cheilocystidia.

##### Etymology.

Epithet “*fulva*” referring to fox-colored pileus.

##### Holotype.

The Republic of Korea, Gyeonggi-do: Pocheon-si, Soheul-eup, Gwangneungsumogwon-ro 415, 37°45'17"N, 127°9'59"E, alt. 101 m, Sang Kook Han, 21 July 2015, KA15–0210 (GenBank accession no. ITS: OL467259; nrLSU: OL462795).

##### Description.

Pileus: 4–20mm, hemispherical, convex to plane, sometimes concave with slightly reflexed, wavy margin, hygrophanous, pale orange (6A3) to greyish orange, becoming more brownish to the center (5B4 to 7C4). Lamellae: distant, *L* = 16–28, *l* = 1–5, sinuate, broad, whitish to yellowish white (4A2) to brownish orange (6C4 to 7C4). Stipe: 7–30 × 0.7–1 mm, cylindrical, gradually widened towards the base, tomentose, apex orange white (5A2) to brownish orange (6C6), becoming dense downwards (6D8), covered with pubescence. Basidiospores: 6.8–9.2 × 3.1–4.9 μm (average 7.47 × 3.69 μm), Q = 2.05, oblong to cylindrical, smooth, colorless, non-dextrinoid, with drops. Basidia: 20.4–29.4 × 4.7–7.8 μm, 4-spored, narrowly clavate, sometimes constricted or curved. Cheilocystidia: (20.5) 24.8–38.4 × 6.5–11.8 μm, lobed, clavate, sometimes with rostrate apex. Pleurocystidia: 31.5–46.9 × 12–20.6 μm, spheropedunculate, obovoid, sometimes with mucronate apex. Trama hyphae: cylindrical to subinflated, irregular, thin-walled, smooth, branched, non-dextrinoid, 2.0‒15 μm wide. Pileipellis: a cutis of cylindrical, thin-walled, 4–15 μm wide hyphae; terminal elements adpressed to suberect, narrowly clavate, thin-walled, with heavy annular ornamentation, 3–8 μm wide. Stipitipellis: a cutis of cylindrical, thin-walled, smooth, 5‒15 μm wide hyphae. Caulocystidia: 45.6–108.3 (131) × 6.8–14.8 μm, cylindrical, irregular, curved. Clamp connections: present in all tissues.

##### Other specimens examined.

The Republic of Korea, Gyeonggi-do: Pocheon-si, Soheul-eup, Gwangneung forest exhibition hall, 37°45'19"N, 127°9'58"E, alt. 99 m, 8 July 2016, Sang Kook Han, KA16–0428. The Republic of Korea, Gyeongsangnam-do: Geochang-gun, Mt. Gibaek, 35°43'6"N, 127°45'49"E, alt. 1095 m, 19 June 2013, Sang Kook Han, KA13–0216.

##### Habit and habitat.

Scattered or gregarious on the bark of deciduous trees or on the rotting branch of both broadleaf trees and conifers, in summer.

##### Distribution.

The Republic of Korea.

##### Remark.

*Collybiopsisfulva* morphologically resembles *Co.menehune* and *Co.ramealis*. They can be distinguished based on several morphological differences. *Collybiopsismenehune* has a longer stipe (15–60 mm length), denser lamellae, and larger basidiospores (7.5–9.5 × 3.5–4.2 μm) (Desjardin et al. 1999). *Collybiopsisramealis* has a smaller basidiocarp (2–20 mm), shorter basidiospores (7.8–11 × 2.5–4 mm) and different type of pileipellis (*Rameales*-structure) (Noordeloos 1983; Desjardin et al. 1997). Phylogenetically, *Co.fulva* is closely related to *Co.ramulicola*. *Collybiopsisramulicola* differs in having a more yellowish pileus, fewer lamellae (9–12) that are brighter in color, a more reddish and thicker stipe (2–3 mm), and smaller sized cheilocystidia (23–27 × 3–6 mm) (Deng et al. 2016).

#### ﻿ Collybiopsis orientisubnuda

Taxon classificationFungiAgaricalesMarasmiaceae

J.S. Kim & Y.W. Lim sp. nov.

8B35FF43-D00F-5AAF-A6A5-5B9AF08F9C84

842056

Fig. 3G–H , Suppl. material 1: Fig. S1D

##### Etymology.

Epithet “*orientisubnuda*” meaning the new species has originated from the East and is morphologically similar to *Co.subnuda*.

##### Holotype.

The Republic of Korea, Gyeongsangbuk-do: Ulleung-gun, 37°31'21"N, 130°53'14"E, alt. 757 m, 19 July 2016, Changmu Kim, Jinsung Lee, Jae Young Park, NIBRFG0000500990 (GenBank accession no. ITS: OL467262; nrLSU: OL546546).

##### Diagnosis.

It features a brownish, 15‒50 mm pileus, orangish cream-colored lamellae, greyish to brownish orange, tomentose, 20–80 × 2.5‒6 mm stipe, subcylindrical to fusoid, 6.7–8.6 × 1.8–3.2 μm basidiospores, and cylindrical, flexuose, sometimes irregular or curved, 26.3–52 (63) × 3.5–6.5 μm caulocystidia. This species is morphologically similar to *Co.subnuda*.

##### Description.

Pileus: 15‒50 mm, convex to plano-convex, sometimes subumbonate; Surface smooth, brownish orange (6C5 to 7C4), becoming paler to the margin (5A2). Lamellae: distant, L = 16–28, l = 3–7, adnexed, pale yellow (4A3) to orange white (5A2). Stipe: 20–80 (100) × 2.5‒6 mm, central to eccentric, cylindrical, tomentose, often twisted, greyish orange (6B4) to brownish orange(7C4), becoming paler and thinner to the base. Basidiospores: 6.7–8.6 × 1.8–3.2 μm (average 7.5 × 2.5 μm), *Q* = 2.5–3.2 (mean = 2.92), cylindrical to fusoid, smooth, hyaline, non-dextrinoid, with drops. Basidia: (17) 19.8–28.7 (29) × 3.7–7.3 μm, 4-spored, narrowly clavate, often constricted. Cheilocystidia: variable in shape and size, 21–33.3 × 4.7–8.2 μm, lobed, clavate, slightly sphaeropendunculate, sometimes constricted or with rostrate apex. Pleurocystidia: 24.7–52.3 × 5.1–9.1 μm, narrowly utriform, clavate, sometimes clavate with rostrate apex. Trama hyphae: cylindrical, often subinflated, smooth, branched, non-dextrinoid, 2.0–7.0 μm wide. Pileipellis: a cutis made up of cylindrical, 2‒8 μm wide hyphae; terminal elements adpressed, cylindrical, often subinflated, with weak annular ornamentation, 3‒6 μm wide. Stipitipellis: a cutis of cylindrical, smooth, 2.5–7 μm wide hyphae. Caulocystidia: 26.3–52 (63) × 3.5–6.5 μm, cylindrical, flexuose, sometimes irregular or curved. Clamp connections: present in all tissues.

##### Other specimens examined.

The Republic of Korea, Chungcheongnam-do: Yesan-gun, Mt. Gaya, 35°48'14"N, 128°5'49”E, alt. 863 m, 23 August 2017, Hae Jin Cho, Ki Hyeong Park, SFC20170823–39. The Republic of Korea, Gangwon-do: Pyeongchang-gun, Mt. Odae, 37°43'54"N, 128°35'42"E, alt. 683 m, 8 July 2017, Nam Kyu Kim, SFC20170708–14. The Republic of Korea, Gyeongsangbuk-do: Ulleung-gun, 37°31'30"N, 130°52'21"E, alt. 718 m, 2 September 2015, Jae Young Park, SFC20150902-01.

##### Habit and habitat.

Scattered to gregarious on the ground covered with dead and decaying leaves of broadleaf forest, from summer to autumn.

##### Distribution.

The Republic of Korea.

##### Remark.

*Collybiopsisorientisubnuda* is morphologically similar to *Co.peronata* (Bolton) R.H. Petersen and *Co.subnuda* (Ellis ex Peck) R.H. Petersen. *Collybiopsisperonata* can be distinguished from *Co.orientisubnuda* by fewer and buff lamellulae (1–3), a thicker stipe (3–8 mm), smaller Q value (2.3), longer basidia (20–40 μm), and longer cheilocystidia (25–90 × 5–10 μm) (Noordeloos et al. 1999). *Collybiopsissubnuda* differs from *Co.orientisubnuda* with thinner stipe (~3 mm), larger basidiospores (8–11 × 3–4.5 μm) and the absence of pleurocystidia (Tekpınar and Acar 2020).

#### ﻿ Collybiopsis subumbilicata

Taxon classificationFungiAgaricalesMarasmiaceae

J.S. Kim & Y.W. Lim sp. nov.

C6CD29F6-3A91-5D89-8AD7-CC600C55C201

842057

Fig. 4A, B , Suppl. material 1: Fig. S1E

##### Etymology.

Epithet “*subumbilicata*” referring to having a small depressed center in pileus.

##### Holotype.

The Republic of Korea, Seoul, Gwanak-gu, Mt. Gwanak, 37°12'39"N, 128°19"E, alt. 877 m, 01 July 2014, Young Woon Lim, SFC20140701–03 (GenBank accession no. ITS: OL467232; nrLSU: OL462787).

##### Diagnosis.

The distinctive features include a brownish, 10–35 mm pileus, white colored lamellae, a brownish, 25–60 × 1‒3 mm stipe covered with pubescence, ellipsoid to oblong basidiospores, narrowly clavate and cylindrical, 17–24.3 × 3.5–5.1 μm basidia, and cylindrical, flexuose, sometimes curved, 12.6–38.2 × 2.4–6.6 μm caulocystidia.

##### Description.

Pileus: 10–35 mm, plano-convex to plano-concave, subumbilicate, becoming undulate and uplifted in age; Surface smooth, greyish orange (5B3) to brown (6E5). Lamellae: subdistant, L = 22–38, l = 3–7, free to adnexed, white. Stipe: 25–60 × 1‒3 mm, cylindrical, tomentose, hollow, light brown (7D4) to dark brown (9F8), becoming paler to the apex, covered with pubescence. Basidiospores: 5.5‒7.5 × 2.5‒3.6 μm (average 6.47 × 3.0 μm), *Q* = 1.8–2.2 (mean = 2), oblong to fusiform, smooth, hyaline, non-dextrinoid, with drops. Basidia: (15.6) 17–24.3 (27.6) × 3.5–5.1 (5.9) μm, 4-spored, narrowly clavate, cylindrical. Cheilocystidia: 17.6–38.4 × 5–7.8 μm, various in shape, lobed. Pleurocystidia: 20.3‒30.7 × 6.8‒9.5 μm, clavate, fusiform, slightly sphaeropedunculate. Trama hyphae: cylindrical, subinflated, branched, smooth, non-dextrinoid, 1.5‒8 μm wide. Pileipellis: a cutis made up of cylindrical, often incrusted, with heavy annular ornamentation, 5.0‒15 μm wide hyphae; terminal elements adpressed to suberect, fusoid, clavate, 6.0‒16 μm wide. Stipitipellis: a cutis of cylindrical, smooth, thin-walled, 2.0‒6.0 μm wide hyphae. Caulocystidia: 12.6–38.2 × 2.4–6.6 μm, cylindrical, flexuose, sometimes irregular or curved. Clamp connections: present in all tissues.

##### Other specimens examined.

The Republic of Korea, Gangwon-do: Goseong-gun, Hwajinpo, Hwajinpo Condominium, 38°28'24"N, 128°26'30"E, alt. 7 m, 2 August 2012, Young Woon Lim, SFC20120802–03. The Republic of Korea, Gyeongsangbuk-do: Ulleung-gun, Ulleung island, 37°30'38"N, 130°51'44"E, alt. 429 m, 22 August 2017, Jae Young Park, Nam Kyu Kim, SFC20170822–14.

##### Habit and habitat.

Scattered to gregarious on the ground covered with dead leaves in temperate mixed forests, from summer to autumn.

##### Distribution.

The Republic of Korea.

##### Remark.

*Collybiopsissubumbilicata* appears similar to *Co.villosipes* (Cleland) R.H. Petersen. *Collybiopsisvillosipes* is distinguished from *Co.subumbilicata* by fewer and brownish lamellae (also lamellulae), a noninsititious, light-colored stipe, larger basidiospores (6.5‒10.5 × 3.5‒4.5 μm) and basidia (25‒34 × 6.5‒7.5 μm) (Desjardin et al. 1997). Furthermore, *Co.subumbilicata* is phylogenetically close to *Co.biformis* and *Co.disjuncta* (R.H. Petersen & K.W. Hughes) R.H. Petersen & K.W. Hughes. *Collybiopsisbiformis* is morphologically similar to *Co.subumbilicata* but can be distinguished by elongated basidiospores (6.4‒9.2 × 2.4‒4.8 μm), thicker basidia (6‒7 μm thick) and cheilocystidia (6‒12 μm thick) (Morgan 1905; Mata 2002). *Collybiopsisdisjuncta* can be distinguished from *Co.subumbilicata* by a smaller pileus (7–12 mm) with olivaceous tint, pinkish lamellae, slender stipe (0.5–1 mm thick), bigger basidiospores (6–7.5 × 3–3.5 μm), bigger basidia (22–34 × 5–7 μm), and a seldom incrusted pileipellis (Petersen and Hughes 2014).

#### ﻿ Collybiopsis undulata

Taxon classificationFungiAgaricalesMarasmiaceae

J.S. Kim & Y.W. Lim sp. nov.

9E693781-2EDA-5E37-B6BE-5FB8E72109E1

842058

Fig. 4C–D , Suppl. material 1: Fig. S1F

##### Etymology.

Epithet “*undulata*” referring to having an undulate margin of pileus.

##### Holotype.

The Republic of Korea, Chungcheongnam-do, Boryeong-si, recreation forest of Mt Sungju, 36°20'4"N, 126°39'50"E, alt. 241 m, 21 August 2012, Jae Young Park, SFC20120821–04 (GenBank accession no. ITS: OL467239; nrLSU: OL462813).

##### Diagnosis.

It is characterized by having 10‒23 mm sized pileus that is particularly brown in the middle with a wavy margin, subdistant and creamy lamellae, a dark brown, 35–55 × 0.8‒2 mm stipe that becomes lighter to the apex, subcylindrical, broadly clavate or irregular, sometimes lobed, 16.7–28 × 4.8–8 μm cheilocystidia, and 27–60 × 3.5–6 μm sized caulocysitida which has a morphology similar to cheilocystidia and sometimes grows in bundles.

##### Description.

Pileus: 10‒23 mm, convex to concave, margin becoming undulate with age; Surface smooth, hygrophanous, brown (7D2 to 7E6) in the center, becoming paler to the margin (5A2–5B3 to 7B2). Lamellae: subdistant, L = 15–30, l = 3–9, adnexed, cream. Stipe: 35–55 × 0.8‒2 mm, cylindrical, tomentose, dark brown (7F5 to 8F8), gradually becoming paler to apex (7B2 to 7C2). Basidiospores: 5.6–9.5 × 2–3.4 μm (average 7.3 × 2.8 μm), Q = 2–3.1 (mean = 2.58), cylindrical, smooth, hyaline, non-dextrinoid, with drops. Basidia: 15–22.3 × 3.6–6.8 μm, 4-spored, cylindrical, narrowly clavate to utriform, often curved. Cheilocystidia: 16.7–28 × 4.8–8 μm, subcylindrical, broadly clavate or irregular, sometimes lobed. Pleurocystidia: absent. Trama hyphae: cylindrical, sometimes subinflated, smooth, branched, non-dextrinoid, 2–8 μm wide. Pileipellis: a cutis made up of cylindrical, often incrusted, slightly brownish, with heavy annular ornamentation, 2.4–7 μm wide hyphae; terminal elements adpressed to suberect, cylindrical to clavate, 3–6 μm wide. Stipitipellis: a cutis of cylindrical, smooth, 2.0‒3.5 μm wide hyphae. Caulocystidia: 27–60 × 3.5–6 μm, irregularly cylindrical, narrowly utriform, seldom apically lobed, sometimes gathered in a bunch. Clamp connections: present in all tissues.

##### Other specimens examined.

The Republic of Korea, Gyeonggi-do: Goyang-si, Deogyang-gu, Seooreung, 37°37'26"N, 126°54'4"E, alt. 35 m, 13 August 2015, Jae Young Park, SFC20150813–04. The Republic of Korea, Gyeongsangbuk-do, Sangju-si, Mt Noheum, 36°26'20"N, 128°5'48"E, alt. 695 m, 8 August 2013, Jae Young Park, SFC20130808–08.

##### Habit and habitat.

Scattered to gregarious on leaf litter in mixed forest dominated with broadleaf trees, in summer.

##### Distribution.

The Republic of Korea.

##### Remark.

*Collybiopsisundulata* is morphologically similar to *Co.subpruinosa* (Murrill) R.H. Petersen. *Collybiopsissubpruinosa* has differences in having small central papilla on pileus, fewer lamellulae (3–4 series), vivid colored lamellae, thicker basidiospores (4.5–5.2 μm wide), larger basidia (30–36 × 7.5–8.5 μm) and cheilocystidia (25–80 × 5–16 μm), thick-walled trama hyphae (0.5–1 μm), caulocystidia with a wider size range, and a habit of growing solitary on rotten twigs or logs (Desjardin et al. 1999). *Collybiopsisundulata* is phylogenetically close to *Co.villosipes* but *Co.villosipes* can be differentiated by having fewer lamelluale (2–3 series), vivid colored lamellae, thicker stipe (1.5–4.0 mm), slightly thicker basidiospores (3.5–4.5 μm wide), and basidia (25–34 × 6.5–7.5 μm) (Desjardin et al. 1997).

#### ﻿ Collybiopsis vellerea

Taxon classificationFungiAgaricalesMarasmiaceae

J.S. Kim & Y.W. Lim sp. nov.

7071A699-E323-52DD-9E3A-3A1D61A9000C

842059

Fig. 4E–F , Suppl. material 1: Fig. S1G

##### Etymology.

Epithet “*vellerea*” refers to having a velvety stipe.

##### Holotype.

The Republic of Korea, Seoul: Gwanak-gu, Mt. Gwanak, 37°27'32"N, 126°56'49"E, alt. 90 m, 21 August 2014, Young Woon Lim, SFC20140821–29 (GenBank accession no. ITS: OL467267; nrLSU: OL462810).

##### Diagnosis.

It has a dull, greyish orange, 18‒45 mm pileus with darker center, a tomentose (like velvet), insititious, orangish, 15–55 × 3‒5 mm stipe, sphaeropendunculate, subovoid, 23.4–49 × 7.5–13.4 μm pleurocystidia, oblong to subcylindrical basidiospores, narrowly clavate with rostrate apex, sometimes lobed, 7.7–49.7 × 3.8–14.6 μm cheilocystidia.

##### Description.

Pileus: 18‒45 mm, hemispherical, appendiculate to convex, subumbonate with an uplifted margin when old; Surface smooth, dull, hygrophanous, orange white (5A2) to greyish orange (6E8 to 7F8) on the center, gradually becoming paler to the edge (5A1 to 5B2). Lamellae: crowded to close, *L* = 38‒52, *l* = 3‒7, furcate, white. Stipe: 15–55 × 3‒5 mm, cylindrical, finely tomentose, insititious, pale orange (5A3) to reddish grey (7B2), becoming darker to the base (6A2 to 7C2). Basidiospores: 5.2‒7 × 2.5‒3.8 μm (average 6.17 × 3.06 μm), *Q* = 1.8–2.4 (mean = 2.03), oblong to subcylindrical, smooth, hyaline, non-dextrinoid, with drops. Basidia: 16.2–24.8 × 3.3–5.3 μm, 4-spored, (narrowly) clavate, often curved or constricted. Cheilocystidia: 7.7–49.7 × 3.8–14.6 μm, narrowly clavate with rostrate apex, sometimes lobed. Pleurocystidia: 23.4–49 × 7.5–13.4 μm, sphaeropendunculate, subovoid. Trama hyphae: cylindrical, often subinflated, thin-walled, smooth, branched, non-dextrinoid, 2‒5 μm wide. Pileipellis: a cutis made up of cylindrical, thin-walled, with weak annular ornamentation, 3‒10 μm wide hyphae; terminal elements adpressed to suberect, cylindrical, fusoid, clavate, 5‒11 μm wide. Stipitipellis: a cutis of cylindrical, thin-walled, smooth, 2.0‒6.0 μm wide hyphae. Caulocystidia: 12–38 × 2.4–6.6 μm, cylindrical, narrowly utriform, sometimes irregular, or curved. Clamp connections: present in all tissues.

##### Other specimens examined.

The Republic of Korea, Chungcheongnam-do: Seosan-si, Mt. Gaya, 36°41'0"N, 126°35'19"E, alt. 260 m, 20 August 2012, Jae Young Park, SFC20120820–02. The Republic of Korea, Incheon: Ongjin-gun, 37°13'10"N, 126°10'4"E, alt. 6 m, 4 September 2018, Changmu Kim, Jin Sung Lee, NIBRFG0000502858. The Republic of Korea, Jeollanam-do: Jindo-gun, Seogeocha island, 34°15'22"N, 125°55'11"E, alt. 38 m, 5 July 2018, Jae Young Park, Tae Heon Kim, SFC20180705–90.

##### Habit and habitat.

Scattered to gregarious on the ground covered with dead and decaying conifer needles, from summer to autumn.

##### Distribution.

The Republic of Korea.

##### Remark.

*Collybiopsisvellerea* is morphologically similar to *Co.menehune* and *G.spongiosus* Halling. *Collybiopsismenehune* has a paler stipe, a smaller pileus (8–30 mm), and fewer lamellulae (4–6 series) (Desjardin et al. 1999). *Gymnopusspongiosus* has a smaller pileus (8–20 mm) and longer stipe (20–55 mm). Micromorphologically, *Co.menehune* has larger basidiospores, basidia, and caulocystidia (Desjardin et al. 1999). *Gymnopusspongiosus* differs from *Co.vellerea* in that its pileipellis is a *Dryophila*-type cutis and its color changes in alkalies. Furthermore, its basidia (18–25 × 6–9 μm) and trama hyphae (3.5–17 μm) are thicker and its caulocystidia (3.5–10.5 μm broad) are smaller (Halling 1996). *Collybiopsisvellerea* is phylogenetically close to *Co.omphalodes*. *Collybiopsisomphalodes* differs in having smaller basidiomata (20–30 mm) and its habit on logs (Dennis 1951).

### ﻿Proposal for *Collybiopsis* recombination

In this study, many epithets were found that required an additional transfer of species from *Marasmiellus* to *Collybiopsis* apart from the study done by Petersen and Hughes (2021). Oliveira et al. (2019) had previously suggested to replace these species from *Gymnopus* to *Marasmiellus* s. str., but this study suggests that these species should be further transferred from *Marasmiellus* s. str. to *Collybiopsis*.

#### ﻿ Collybiopsis istanbulensis

Taxon classificationFungiAgaricalesMarasmiaceae

(E.Sesli, Antonín & E.Aytaç) J.S. Kim & Y.W. Lim comb. nov.

BC7F3415-98EC-58CA-B3C3-896F2AA95A0B

842060

##### Basionym.

*Marasmiellusistanbulensis* E. Sesli, Antonín & E.Aytaç. Pl. Biosystems 152(4): 669. 2018.

#### ﻿ Collybiopsis koreana

Taxon classificationFungiAgaricalesMarasmiaceae

(Antonín, Ryoo & H.D.Shin) J.S. Kim & Y.W. Lim comb. nov.

A90B99B1-5939-5F44-B776-642378DFA320

842061

##### Basionym.

*Marasmielluskoreanus* Antonín, Ryoo and H.D.Shin. Mycotaxon 112: 190. 2010.

#### ﻿ Collybiopsis omphalodes

Taxon classificationFungiAgaricalesMarasmiaceae

(Berk.) J.S. Kim & Y.W. Lim comb. nov.

1A99ECC0-7395-5CE6-8327-888E04B4C870

842062

Chamaeceras omphalodes (Berk.) Kuntze, Revis. gen. pl. (Leipzig) 3(3): 456. 1898.
Collybia omphalodes (Berk.) Dennis, Trans. Br. mycol. Soc. 34(4): 443. 1951.
Marasmiellus omphalodes (Berk.) Singer. Sydowia 9(1–6): 385. 1955.
Gymnopus omphalodes (Berk.) Halling & J.L. Mata, in Mata, Halling, and Petersen, Fungal Diversity 16: 122. 2004.

##### Basionym.

*Marasmiusomphalodes* Berk., Hooker’s J. Bot. Kew Gard. Misc. 8: 138. 1856.

#### ﻿ Collybiopsis pseudomphalodes

Taxon classificationFungiAgaricalesMarasmiaceae

(Dennis) J.S. Kim & Y.W. Lim comb. nov.

033C67C2-3FC7-5C8A-B8A3-87BD04E400EF

842063

Gymnopus pseudomphalodes (Dennis) J.L. Mata, in Mata, Hughes, and Petersen, Sydowia 58(2): 289. 2006, as “pseudo-omphalodes”.
Marasmiellus pseudomphalodes (Dennis) J.S. Oliveira, in Oliveira, Vargas-Isla, Cabral, Rodrigues and Ishikawa, Mycol. Progr. 18(5): 735. 2019, as “pseudomphalioides”.

##### Basionym.

*Collybiapseudomphalodes* Dennis, Kew Bull. 15(1): 74 (1961).

#### ﻿ Collybiopsis ramulicola

Taxon classificationFungiAgaricalesMarasmiaceae

(T.H. Li & S.F. Deng) J.S. Kim & Y.W. Lim comb. nov.

716030F4-1567-5AD9-81B0-051497F06219

842064

##### Basionym.

*Gymnopusramulicola* T.H. Li & S.F. Deng, in Deng, Li, Jiang and Song, Mycotaxon 131(3): 665. 2016.

### ﻿Taxonomic key to *Collybiopsis* in Korea

**Table:** 

1	Pileus < 25 mm diam	**2**
–	Pileus > 25 mm diam	**11**
2	Lamellae subdistant to distant (10–30)	**3**
–	Lamellae close to crowded (> 30)	**9**
3	Basidiomes on bark, branch, or woody debris	**4**
–	Basidiomes on duff or on soil	**8**
4	Pleurocystidia present	**5**
–	Pleurocystidia absent	**7**
5	Stipe base covered with dense whitish basal tomentum	**Co.albicantipes**
–	Stipe base not covered with whitish basal tomentum	**6**
6	Pileipellis composed of a coarse *Rameales*-structure hyphae	**Co.ramealis**
–	Pileipellis composed of a cylindrical, often sub-inflated hyphae, not a Rameales-structure	**Co.fulva**
7	Pileus distinctly sulcate. Stipe base covered with dense whitish basal tomentum	**Co.koreana**
–	Pileus slightly sulcate. Stipe base covered with weak whitish basal tomentum	**Co.nonulla**
8	Stipe < 2 cm long. Q value of basidiospores 1.6–2.2	***Co.dichroa***
–	Stipe > 2 cm long. Q value of basidiospores 2.0–3.1	**Co.undulata**
9	Lamellae crowded (> 100)	***Co.confluens***
–	Lamellae close to crowded (< 100)	**10**
10	Basidia > 22 µm long	***Co.menehune***
–	Basidia < 22 µm long	**Co.biformis**
11	Lamellae subdistant to distant (10–38)	**12**
–	Lamellae close (> 38)	**15**
12	Pleurocystidia present	**13**
–	Pleurocystidia absent	**14**
13	Q value of basidiospores > 2.2	**Co.orientisubnuda**
–	Q value of basidiospores < 2.2	**Co.subumbilicata**
14	Pileus convex, hemispherical, plano-convex to flat. Cheilocystidia utriform and clavate	***Co.clavicystidiata***
–	Pileus convex to broad-convex. Cheilocystidia narrowly clavate	***Co.polygramma***
15	Pleurocystidia present	***Co.vellerea***
–	Pleurocystidia absent	**Co.luxurians**

## ﻿Discussion

Of the 372 gymnopoid/marasmioid specimens, we confirmed 201 specimens (54%) to belong to *Collybiopsis*. These results indicate that the species of *Collybiopsis* can be confused with those of similar genera as well as with other *Collybiopsis* members when identification is based solely on morphological information. This is because some characteristics are overlapped between species (Suppl. material 2: Fig. S2) and the characteristics can be different depending on developmental stage or environmental conditions. Further, the high misidentification ratio may be caused by the slow rate of adoption of the current names. Sequence-based taxonomy has introduced rapid changes in the classification of gymnopoid/marasmioid species (Mata 2002; Mata et al. 2004a; Mata et al. 2004c; Hughes et al. 2010; Oliveira et al. 2019; Petersen and Hughes 2017, 2021). As such, taxonomic confusion has been resolved in taxa that have been well researched based on molecular data (Desjardin et al. 1999; Mata 2002; Lee et al. 2019).

Nine of the sixteen *Collybiopsis* species were identified as already known species. Of the nine described species, seven species were identified as the species previously recorded in the Republic of Korea: *Collybiopsisbiformis*, *Co.confluens*, *Co.koreana*, *Co.luxurians*, *Co.menehune*, *Co.polygramma*, and *Co.ramealis*. Two species, *Co.dichroa* and *Co.nonnulla*, were reported for the first time in the Republic of Korea. Most of the nine described species formed a monophyletic clade with each corresponding species. However, sequence variations by continent were detected in *Co.biformis*, *Co.confluens*, *Co.dichroa*, and *Co.nonnulla*. Asian samples, including our specimens, were clearly separated from those of Europe, North America, and Africa. These results have also been reported in previous studies on *Collybiopsisbiformis* (Mata 2002; Petersen and Hughes 2014; Razaq et al. 2020) and *Co.confluens* (Hughes and Petersen 2015). Especially, *Co.confluens* is known as a representative example of intra-specific variation between continents. Percent ITS sequence divergence of this species was reported to be 3.25% when comparing the sequences of the North America and Europe (Hughes and Petersen 2015). We confirmed that percent ITS sequence divergence of Asian *Co.confluens* (our Korean samples and Chinese sequences) were each about 3% when compared to American and European sequences.

Similarly, *Co.dichroa* showed sequence variations that were previously reported in association with intraspecific hybridization and dramatic sequence variations including frequent nucleotide substitutions of Adenine and Guanine (Hughes et al. 2015). The Korean *Co.dichroa* was closely related to *Co.dichroa* taxa 2 mentioned in Hughes et al. 2015. Similarly, the intraspecific genetic variation depending on environmental conditions or geographical distribution has been reported in many other fungal species (Manian et al. 2001; Kauserud et al. 2007; Seierstad et al. 2013). For the last, Korean *Co.nonnulla* showed high intra-specific divergence when matching with sequences of *Co.nonnulla* of America and Cameroon. According to the phylogenetic analysis results, there is a slight sequence variation, but it forms a clade supported by a high bootstrap and morphologically almost coincides with the reference. Therefore, we view this sequence variation as due to different environments by continent and identify the specimens as *Co.nonnulla*. Nevertheless, compared to the fact that it was reported as a new species a long time ago, only seven sequences were deposited in the NCBI, so further study on this species is necessary.

Morphologically, the morphological characteristics of the seven described species were also in agreement with the previous descriptions (Suppl. material 2: Fig. S2). However, *Co.luxurians* and *Co.polygramma* found in the Republic of Korea showed few differences compared to the Western descriptions in the previous literature (Mata 2003; Noordeloos et al. 1999). In the case of the *Co.luxurians*, Korean sequences formed a slightly distinct clade in the phylogenetic tree, along with the Chinese sequence (ZD16102301), from European sequences. In this study, direct morphological comparison studies with European and Chinese samples were difficult and there was no significant morphological difference from the references. For these reasons, we identified Korean specimens as *Co.luxurians*, but further studies are needed with more samples from other countries for this species.

Seven new species have common characteristics of *Collybiopsis* such as insititious to subinsititious stipe, ellipsoid to oblong, inamyloid basidiospores, and presence of caulocystidia. However, it is difficult to distinguish them from other *Collybiopsis* species based on morphological characteristics alone. Upon molecular phylogenetic analyses, each of them clearly formed a distinct clade clearly in the ML phylogenetic tree (Fig. 1). Their morphological features may or may not be distinguished from their phylogenetically close relatives. The morphological differences between new species and morphologically similar or phylogenetically close species are discussed in the remarks for each species.

Two species previously reported in the Republic of Korea, *Co.peronata* (Cho & Lee, 1979) and *Co.subnuda* (National list of species of Korea 2020), were not confirmed in this study. *Co.peronata* and *Co.subnuda*, which are typical collybioid mushrooms, have been reported in Asia based on their morphological characteristics (Cho and Lee 1979; Kim et al. 1991; Park and Cho 1992; Yoshida and Muramatsu 1998; Tolgor and Yu 2000). Molecular analyses showed that none of the Korean specimens examined in this study could be identified as *Co.peronata* nor *Co.subnuda*. Instead, the specimens labelled as *Co.peronata* or *Co.subnuda* were identified as different species – *Gymnopussimilis* Antonín, Ryoo & Ka and *Co.orientisubnuda*. *Collybiopsisperonata* were originally mostly reported from Europe and America and *Co.subnuda* were originally reported from America (Desjardin et al. 1999; Mata et al. 2006). Furthermore, there have been no recent sequence uploads to GenBank or reports of *Co.peronata* and *Co.subnuda* from Asia, making it difficult to confirm whether they exist in the Republic of Korea. Although *Co.orientisubnuda* is closely related to *Co.peronata* and *Co.subnuda*, there are clear differences in the ITS regions of these three species (Suppl. material 3: Fig. S3). Morphologically, *Co.orientisubnuda* is highly similar to *Co.subnuda* and considerably different from *Co.peronata*. The detailed comparisons of the morphological features are provided in the remarks for each species.

In conclusion, we identified 16 *Collybiopsis* species in the Republic of Korea through morphological and molecular analyses and we update the Korean inventory of *Collybiopsis*. Our study showed that the identification of *Collybiopsis* species requires both morphological and molecular analyses. Further, this study has the following significance as in the previous study conducted by Petersen and Hughes (2021): additional combinations of *Marasmiellus* species under *Collybiopsis*, detailed morphological characterization of *Collybiopsis* species in the Republic of Korea along with photographs and drawings, and specific approaches to species differentiation and identification through morphological and molecular analyses. Furthermore, we believe that this study will be helpful for further studies such as research of *Collybiopsis* distribution worldwide as it provides additional molecular information about *Collybiopsis* in the Republic of Korea and proposes seven new species identified from the Republic of Korea. These data will be useful for the identification and taxonomic arrangement of gymnopoid/marasmioid mushrooms.

## Supplementary Material

XML Treatment for Collybiopsis albicantipes

XML Treatment for Collybiopsis clavicystidiata

XML Treatment for Collybiopsis fulva

XML Treatment for Collybiopsis orientisubnuda

XML Treatment for Collybiopsis subumbilicata

XML Treatment for Collybiopsis undulata

XML Treatment for Collybiopsis vellerea

XML Treatment for Collybiopsis istanbulensis

XML Treatment for Collybiopsis koreana

XML Treatment for Collybiopsis omphalodes

XML Treatment for Collybiopsis pseudomphalodes

XML Treatment for Collybiopsis ramulicola

## Supplementary materials

Supplementary material 1 Figure S1 Data type Jpg file. Explanation note Pileipellis elements of seven new species. **A***Collybiopsisalbicantipes***B***Co.clavicystidiata***C***Co.fulva***D***Co.orientisubnuda***E***Collybiopsissubumbilicata***F***Co.undulata***G***Co.vellerea*. Scale bars: 10 *µm*. https://binary.pensoft.net/file/666089This dataset is made available under the Open Database License (http://opendatacommons.org/licenses/odbl/1.0/). The Open Database License (ODbL) is a license agreement intended to allow users to freely share, modify, and use this Dataset while maintaining this same freedom for others, provided that the original source and author(s) are credited.Ji Seon Kim, Yoonhee Cho, Ki Hyeong Park, Ji Hyun Park, Minkyeong Kim, Chang Sun Kim, Young Woon Lim

Supplementary material 2 Figure S2 Data type Jpg file. Explanation note Comparison of the morphological characters of 16 Korean *Collybiopsis* species. https://binary.pensoft.net/file/666090This dataset is made available under the Open Database License (http://opendatacommons.org/licenses/odbl/1.0/). The Open Database License (ODbL) is a license agreement intended to allow users to freely share, modify, and use this Dataset while maintaining this same freedom for others, provided that the original source and author(s) are credited.Ji Seon Kim, Yoonhee Cho, Ki Hyeong Park, Ji Hyun Park, Minkyeong Kim, Chang Sun Kim, Young Woon Lim

Supplementary material 3 Figure S3 Data type Jpg file. Explanation note Sequence difference between the three species in the ITS region. https://binary.pensoft.net/file/666091This dataset is made available under the Open Database License (http://opendatacommons.org/licenses/odbl/1.0/). The Open Database License (ODbL) is a license agreement intended to allow users to freely share, modify, and use this Dataset while maintaining this same freedom for others, provided that the original source and author(s) are credited.Ji Seon Kim, Yoonhee Cho, Ki Hyeong Park, Ji Hyun Park, Minkyeong Kim, Chang Sun Kim, Young Woon Lim
