# Supplementary figures and images for: ﻿Taxonomic study of Collybiopsis (Omphalotaceae, Agaricales) in the Republic of Korea with seven new species
Source: MycoKeys. 2022 Mar 30;88:79–108. doi: 10.3897/mycokeys.88.79266 (PMC9005495; doi:10.3897/mycokeys.88.79266)

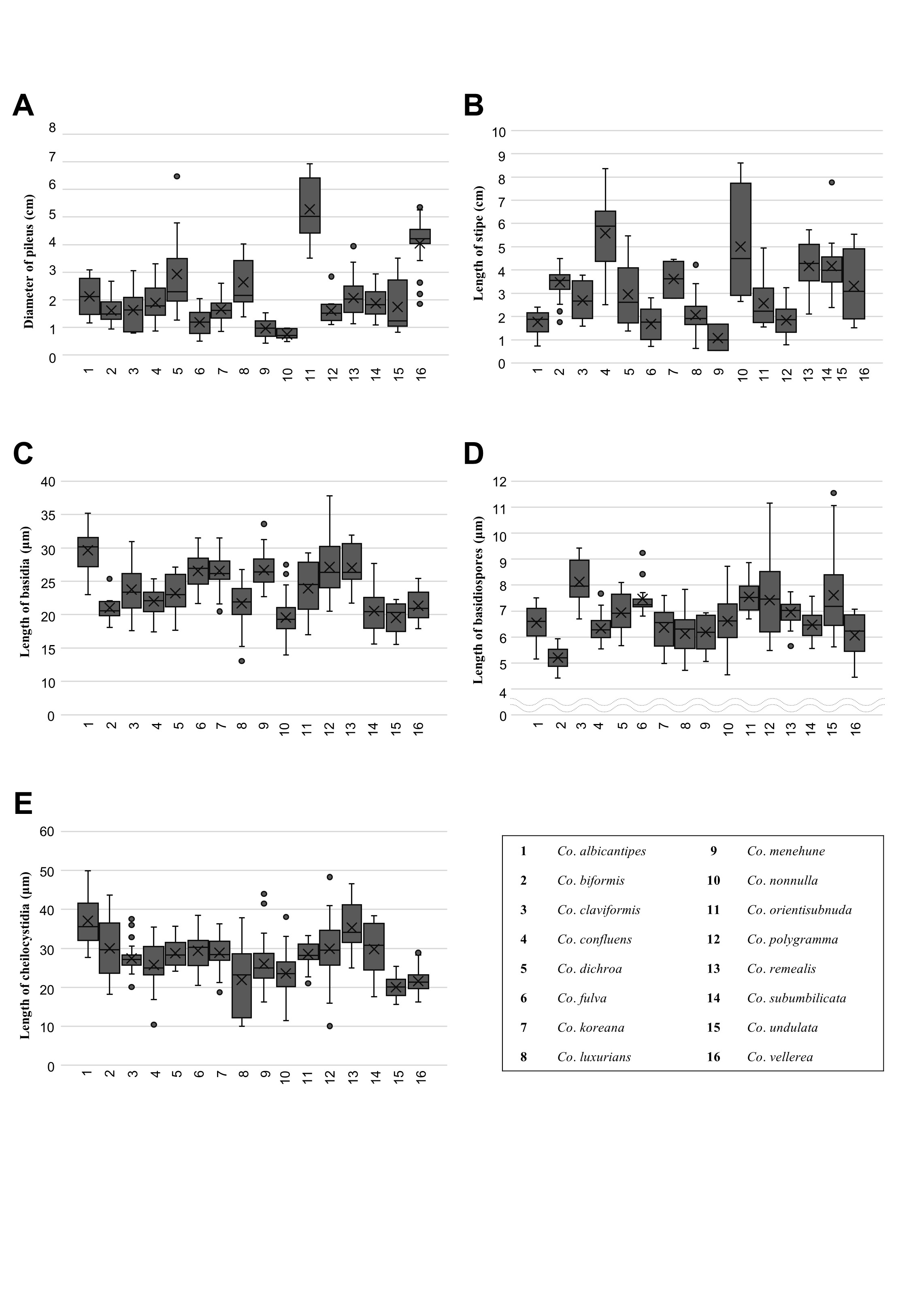

Supplement: Supplementary material 1 — Figure S1 [file mycokeys-88-079-s001.jpg]

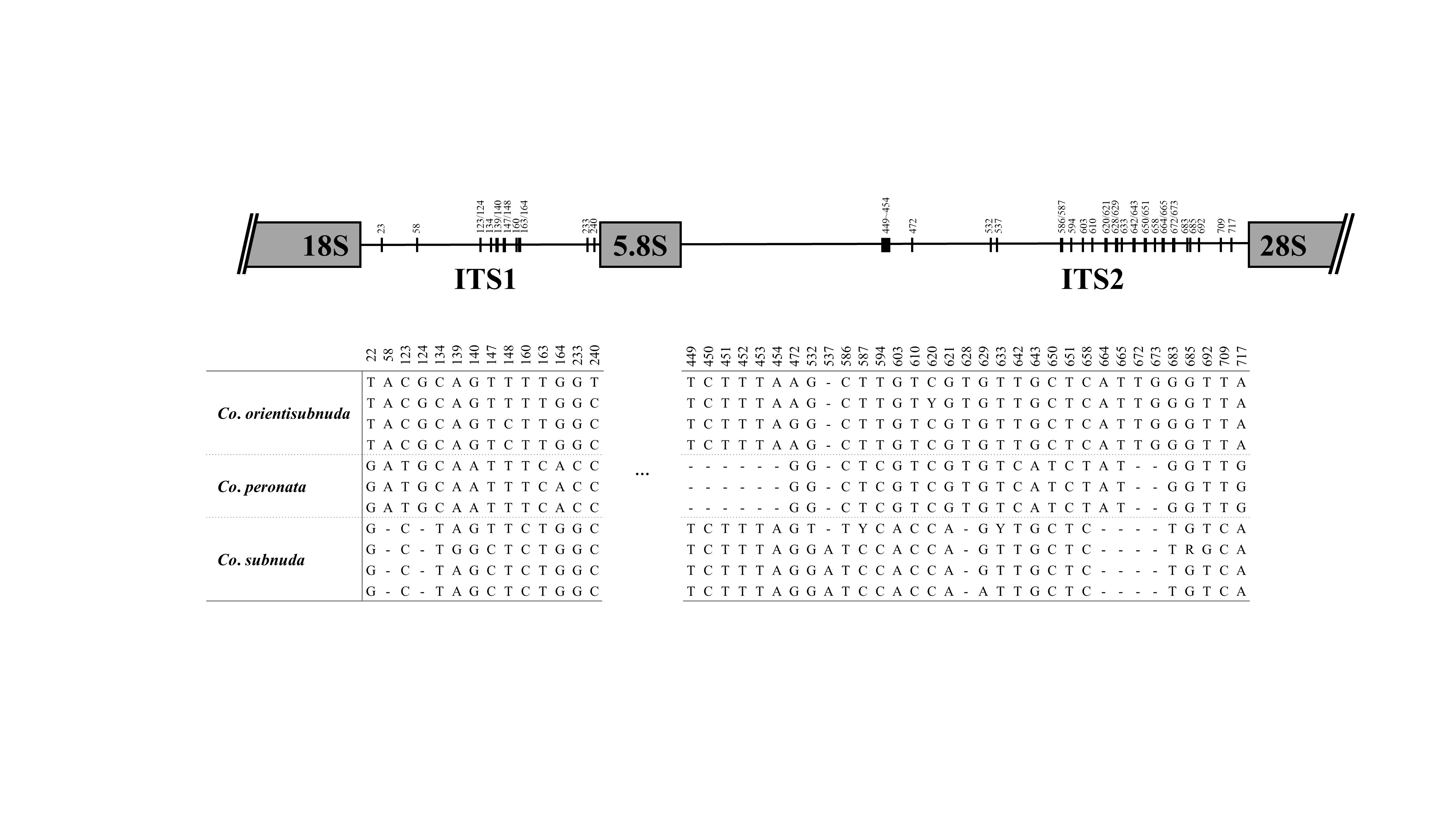

Supplement: Supplementary material 2 — Figure S2 [file mycokeys-88-079-s002.jpg]

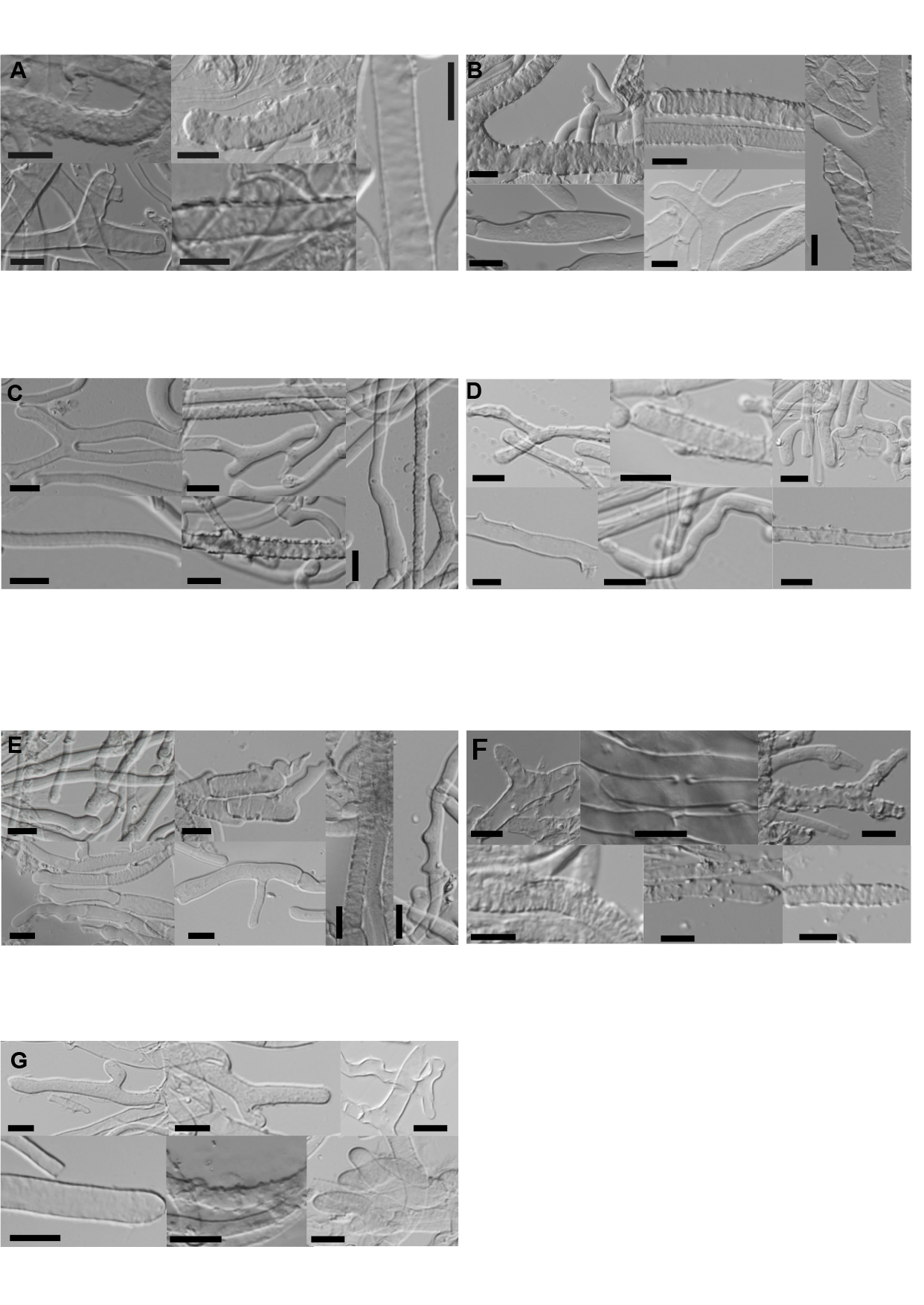

Supplement: Supplementary material 3 — Figure S3 [file mycokeys-88-079-s003.jpg]
